# Development and Investigation of a New Model Explaining Job Performance and Uncertainty among Nurses and Physicians

**DOI:** 10.3390/ijerph18010164

**Published:** 2020-12-28

**Authors:** Espen Olsen, Aslaug Mikkelsen

**Affiliations:** 1 Department of Innovation, Leadership and Marketing, UiS Business School, University of Stavanger, 4036 Stavanger, Norway; aslaug.mikkelsen@uis.no; 2 Stavanger University Hospital, 4011 Stavanger, Norway

**Keywords:** management, leadership, psychological needs, healthcare services, job performance, uncertainty, job resources

## Abstract

The purpose of this paper is to develop and investigate a new theoretical model explaining variance in job performance and uncertainty among nurses and physicians. The study adopted a cross-sectional survey. Data was collected from 2946 nurses and 556 physicians employed at four public hospitals in Norway. We analysed data using descriptive statistics, correlations, Cronbach’s alpha, confirmatory factor analyses and structural equation modelling. To explain job performance and uncertainty, two sets of explanatory variables were used: first, satisfactions of three psychological needs—namely autonomy, social support and competence development—and second, employee perceptions of hospital management quality (HMQ) and local leadership quality (LLQ). The results supported the theoretical model among nurses and physicians; (1) HMQ was positively associated with LLQ; (2) LLQ was positively associated with psychological needs; (3) the majority of psychological needs were positively associated with job performance and negatively associated with uncertainty, but more of these relations were significant among nurses than physicians. The results suggest that job performance and uncertainty among nurses and physicians can be improved by helping personnel meet their psychological needs. Improving job design and staff involvement will be important to strengthen need satisfaction. Results suggest enhancement of HMQ and LLQ will be positively related to need satisfaction among nurses and physicians and will strengthen job performance and reduce uncertainty.

## 1. Introduction

The complexity of healthcare systems, tasks and patient care can develop high levels of uncertainty among healthcare workers. In virtually all clinical situations experienced by patients and health professionals, uncertainty is interwoven on a daily basis. Uncertainty is influenced by numerous unknowns. Will a patient develop a particular condition? How will that condition evolve? Is the treatment beneficial? Is the patient receiving the right care at the right time, in the right place, and from the right people? Hence, the variety of these unknowns, behaviours and feelings, reflects the concept of uncertainty and “make uncertainty a ubiquitous problem in health care” [1]. Uncertainty influences how people think, feel or behave [1]. Moreover, based on the rapid emergence and development of new medical technologies, uncertainty is a growing problem in healthcare. Uncertainty has many potential psychological effects and is a critical phenomenon in healthcare. Uncertainty can provoke fear, worry, anxiety and avoidance of decision-making. Individuals will, therefore, engage in a variety of different responses to minimise the negative effects of uncertainty. Some will try to avoid uncertainty, while others will seek information to reduce uncertainty. The responses to uncertainty will also depend on the specific context [1,2]. Given the increasing exposure of health providers to uncertain situations and information, researchers should develop knowledge that can be used to reduce uncertainty and improve uncertainty tolerance in healthcare. For this to happen, we need research that can increase the general understanding of uncertainty in healthcare settings [1], a clearer understanding of the causes of uncertainty, and new response strategies [3].

Many factors are related to uncertainty among clinicians, and this study aims to develop a taxonomy explaining direct and indirect causes of uncertainty and job performance among nurses and physicians. Since many factors at different levels influence clinicians, both micro- and macro-organisational factors will be included in the development of a theoretical model. First, we assume that both managers and leaders influence the work setting and psychological need satisfaction among clinicians. Moreover, we assume that psychological need satisfaction significantly influences uncertainty and job performance. In summary, we use these assumptions to develop a new taxonomy linking hospital management quality (HMQ), local leadership quality (LLQ), psychological need satisfaction, uncertainty and job performance. This new taxonomy is to be tested among nurses and physicians. The study provides new insights into the system dynamics of hospitals, starting from the top management down to each individual worker who treats patients in clinical settings. Furthermore, the study aims to contribute new knowledge that can be used to understand clinical mechanisms and to improve hospital performance and delivery of care in various settings. To cross-validate the research findings and potentially explore unique findings across different groups, hypothesis testing will be conducted separately for nurses and physicians. 

Psychological health has a moderate to strong correlation with job performance across scientific studies [4]. Indisputably, healthcare institutions aim to avoid burnout of staff and develop healthy working conditions that support job performance. Unfortunately, healthcare institutions are often characterised as the opposite, with working conditions poorer than other sectors’ increasing the likelihood of burnout and reduced quality of care [5]. 

Many factors influence system outcomes and quality of care. Hence, it may be necessary to integrate individual factors with micro- and macro-organisational factors when trying to understand systems’ dynamics [6]. Both managers and leaders influence the work context of healthcare staff [7], and research suggests that the work environment significantly influences patient care [8,9,10]. In the current study, we take these principles into account. Therefore, we develop and present a new theoretical model illustrating how hospital management quality (HMQ) is positively related to local leadership quality (LLQ). Furthermore, LLQ of hospital wards is expected to be positively related to the satisfaction of employees’ important psychological needs, namely autonomy, competence development and colleague support [11]. Since delivery of care is mediated by the performance of each individual healthcare worker, we also suggest that fulfilling the psychological needs of hospital employees has the potential to improve job performance and reduce uncertainty among staff. Hence, the current study focuses on incorporating and integrating important system components influencing healthcare workers’ delivery of care. A new holistic framework is developed which integrates HMQ and LLQ with the level of need satisfaction, which in turn is expected to improve job performance and reduce uncertainty. This framework will be tested with survey data collected from nurses and physicians. Furthermore, we will test the validity of a structural model reflecting the theoretical framework, validating this model based on data. 

### 1.1. Theoretical Framework and Hypotheses

**Figure 1:**
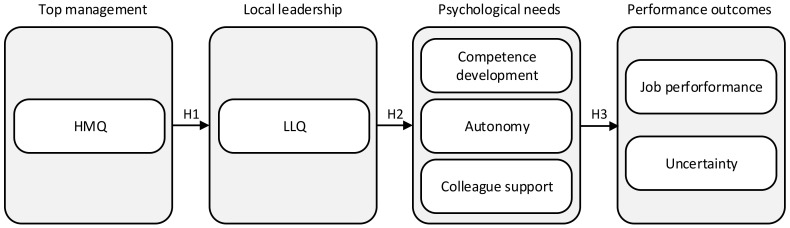
Research model. HMQ: Hospital management quality; LLQ: Local leadership quality.

This study draws upon theories originating from various bodies of literature. First, using knowledge from leadership and management literature, HMQ and LLQ are conceptualised and theoretically linked to each other. Next, insights from self-determination literature are used to conceptualise how HMQ and LLQ are linked to the satisfaction of psychological needs of healthcare personnel. Finally, theoretical arguments are built to hypothesise two model outcomes: uncertainty and job performance (Figure 1). Hence, we combine insights from management and leadership theory with organisational psychology and apply these sources to study uncertainty and job performance among physicians and nurses in a hospital context. 

#### 1.1.1. Managers’ Influence on Leaders

Management and leadership have been substantially studied for decades, have multiple approaches and definitions [12], and have been empirically linked to outcomes both in healthcare [8,13,14] and other industries, e.g., [15].In the current study, we focus on core areas of leadership and management: HMQ and LLQ. Hospital management must cope with financial pressures and conflicting demands while dealing with pressure from the board. Research suggests that managers select different coping strategies to handle conflicting values and that these mechanisms challenge the integrity of managers [16] as well as their choices and priorities. Furthermore, to correctly prioritise, hospital managers need to have knowledge at both the institutional and regional levels. Hence, we suggest that HMQ consists of the following managerial skills: (1) correct prioritisation, (2) adequate knowledge at the institutional level, and (3) knowledge at the regional level which constitutes the hospital context.

Hospital managers define a large share of hospital agendas and implement prioritisations top-down in hospital organisations [17]. On the other hand, leaders at lower levels must potentially handle pressure from staff, patients and next of kin. In the daily care of patients; therefore, it is likely that the perception of HMQ and LLQ will vary across staff. Specifically, we include three leadership elements we regard as most important related to LLQ: (1) relational skills to retain workers, (2) overview and knowledge at the local hospital level, and (3) the ability to develop employees. Summarised, we expect HMQ to strengthen LLQ, which leads to the following hypothesis:

**H1**: *HMQ will be positively related to LLQ.*

#### 1.1.2. Leadership Is Related to Psychological Needs

Next, we consider the potential positive influence LLQ has on the satisfaction of hospital staff’s psychological needs. According to the self-determination theory, relatedness and inclusion in social groups constitute an important psychological need [18]. Leaders have the potential to build, develop and influence the social relationships in wards and units. Developing relationships is a potential way in which managers can influence perceived social belonging among employees. Healthcare professionals need to interact with one another as well as with professionals in other fields, developing mutual respect that will positively affect the work environment and social support [18]. 

Another topic concerns the potential influence leaders have on the need for autonomy. Research suggests the leaders have the potential to empower employees [19]. We, therefore, expect that managers can have a great effect on the autonomy of workers by either restricting or increasing it. The control that local managers have over different aspects of the workplace considerably influences the ways in which the employees perceive their work environment as controlling versus autonomous, which, according to self-determination theory, is important for motivation [20]. An independent and autonomous employee will have the opportunity to express what needs to be done, how and when. An employee may still autonomously complete a task that has been assigned by a supervisor as long as the employee believes that the nature of the task is inherently interesting and congruent with his or her values [21]. A leader may support autonomy through empowerment strategies, through sharing control over how the work gets done, and through trying to understand employees’ perspectives on the work [19,22].

The need for and development of competence is the third important psychological need, according to Deci and Ryan [20]. We expect that managers have the capability to satisfy the need for competence by delegating tasks that fit well with individual employees’ skills and abilities and by developing such skills and abilities according to goals, tasks and patient treatment challenges. Earlier research suggests that a transformational leadership style is positively related to competence, while management by exception is negatively related to competence [11]. 

Baard et al. [22] found that leadership behaviours that promote the satisfaction of employees’ basic psychological needs produced positive outcomes, such as motivation, whereas behaviours that prevented need satisfaction led to negative outcomes. In summary, we expect LLQ to be positively related to employees’ need satisfaction of autonomy, competence development and support [20]. Accordingly, the following hypothesis was formulated:

**H2**: *LLQ is positively related to psychological need (competence development, colleague support and autonomy) satisfaction.*

#### 1.1.3. Psychological Needs Are Related to Uncertainty and Job Performance

Next, we will examine the potential outcomes of satisfying employees’ psychological needs. Various studies have indicated that when people’s basic psychological needs are satisfied, they behave with a sense of willingness and choice, e.g., [23]. Such positivity and engagement among staff have the potential to improve patient care [24,25,26]. On the contrary, lower engagement reflected in burnout is expected to be negatively related to patient safety and satisfaction outcomes [27,28,29]. 

Satisfaction of the three basic psychological needs is expected to positively influence health and well-being [30,31] as well as performance [22,32]. Individual job performance consists of distinct sets of activities that contribute to an organisation’s output. When employees have satisfied their needs for competence, autonomy and social support, they will be motivated to invest their physical, cognitive and emotional energies into their work, e.g., [20,33,34,35]. This, again, may enhance job performance among hospital staff. Furthermore, by satisfying employees’ need for autonomy, competence and relatedness, leaders are creating an environment where all employees can perform better, with positive emotions and higher engagement levels [36]. The research connecting need satisfaction to well-being and high-quality performance has been demonstrated in many fields [35], including healthcare [34].

Meta-analyses [37] of organisational studies suggest that poor worker health, e.g., as exemplified by burnout, is positively associated with adverse events and accidents. On the other hand, job resources, such as autonomy and social support, are related to less burnout and higher job engagement [37]. Transferring findings from the meta-analysis, we expect need satisfaction to be positively related to job performance and negatively related to uncertainty. Therefore, we expect that the fulfilment of psychological needs will be negatively related to uncertainty and positively related to job performance. This leads to the following hypothesis:

**H3**: *Satisfaction of psychological needs are (a) negatively related to uncertainty and (b) positively related to job performance.*

#### 1.1.4. Final Model to Be Tested

This study contributes to the development and testing of a theoretical framework illustrated in Figure 1. This framework explains the direct and indirect links from hospital top management (HMQ) and local leadership (LLQ) to psychological need satisfaction, which is related to two outcomes in hospitals: uncertainty and job performance. A multilevel organisational perspective is integrated into the model, suggesting that managers indirectly influence the psychological needs of hospital employees and that local leaders directly influence need satisfaction. To cross-validate the research findings and potentially explore unique findings across different groups, hypothesis testing is conducted separately for nurses and physicians.

## 2. Material and Methods

### 2.1. Sample and Data Collection

The current study was carried out in a Norwegian health region providing services to a population of 1.1 million citizens. The human resource departments across the hospitals generated employee email listings. A cross-sectional web-based survey design was employed, and 22,883 employees working across four hospitals in the health region were engaged. The overall response rate was 40% (*N* = 9162). For the purpose of this study, physicians (*N* = 556) and nurses (*N* = 2946) were selected from the total sample. Hence, 3502 respondents were included in the current study.

### 2.2. Measures

All study variables were based on employees’ perception, with the use of Likert type scales. All measurement concepts were operationalised with the use of multi-item measures.

Hospital management quality (HMQ) and local leadership quality (LLQ) were adopted and developed based on earlier studies using the Work Research and Quality Improvement Questionnaire [38,39,40].

HMQ was measured with three items using a five-point scale (1 = not correct, 5 = totally correct). The items assessed whether hospital management had knowledge about departments, whether management priorities were correctly based on holistic understanding, and whether regional management possessed strong knowledge about the hospital. An example item reads, “The hospital management has good knowledge about the work in the different departments.” Cronbach’s alpha was 0.84.

LLQ was measured using three items on a five-point scale (1 = not correct, 5 = totally correct). The items assessed the leaders’ ability to retain workers, to possess general knowledge, and to develop employees. An example item reads, “My closest leader has good knowledge about my working situation.” Cronbach’s alpha was 0.86.

Autonomy [41] was based on an index consisting of four items measured on a five-point scale (1 = to a very small extent, 5 = to a great extent). An example item reads, “Employees have good opportunities to influence how work is carried out.” Cronbach’s alpha was 0.91.

Competence development [42] was assessed with four items on a five-point scale (1 = to a very small extent, 5 = to a great extent). An example item reads, “Do you have the opportunity to learn new things through your work?”. Cronbach’s alpha was 0.76. 

Colleague support [43] was assessed with three items on a five-point scale (1 = never, 5 = very often). An example item being “Are your colleagues able to appreciate the value of your work and see the results of it?”. Cronbach’s alpha was 0.74.

Job performance [44] comprised four items measured on a five-point scale (1 = very seldom/never, 5 = very often/always). An example item reads, “Are you satisfied with the quality of the work you carry out?”. The other items concerned their ability to solve problems at work, capacity to maintain good working relationships with colleagues, and satisfaction related to the amount of work conducted. Cronbach’s alpha was 0.77.

Uncertainty comprised six items measuring job-related situations associated with uncertainty on a four-point scale (1 = never, 4 = very often). Perception of uncertainty was, for instance, related to insufficient information and doubt regarding whether patients’ relatives should be informed of patients’ medical condition and treatment. The items were adopted from the Nurses Early Exit Study (http://www.next-study.net). An example item reads, “Uncertainty regarding the use and function of special equipment.” Cronbach’s alpha was 0.65.

### 2.3. Data Analysis

Descriptive statistics and multivariate analysis of variance (MANOVA) were performed with SPSS 26.0, while the remaining assessments were performed using AMOS 25.0. Pearson’s correlations indicated some overlap between concepts. Since social phenomena were expected to vary across groups, MANOVA (Wilks’ lambda) was conducted to test differences between the means of identified groups of subjects on a combination of variables. Variance across demographic variables should be expected and should support the discriminant validity of the study.

Confirmatory factor analyses (CFA) with the use of maximum likelihood estimation (MLE) was performed to test the validity of constructs. All the latent variables and observed variables were entered simultaneously to assess the construct validity. CFA is important since survey instruments are evolving, and the validity of measurements may differ across contexts. Second, structural equation modelling (SEM) with the use of MLE was performed to test the hypothetical model developed. The following indicators and thresholds were used to evaluate the fit: the root mean square error of approximation (RMSEA), Tucker–Lewis Index (TLI), incremental fit index (IFI > 0.90) and comparative fit index (CFI > 0.90). An RMSEA of less than 0.05 indicates a “good” fit, and an RMSEA of less than 0.08 corresponds to an “acceptable” fit [45]. Values of 0.90 or greater for other indicators indicate a “good” fit [45,46]. Chi-square is normally not recommended to evaluate fit for larger samples and was, therefore, not employed [47].

Based on the two target groups selected for the current study, descriptive statistics, CFA and structural modelling were run separately for nurses and physicians to cross-validate findings.

## 3. Results

### 3.1. Demographics

A total of 2946 nurses and 556 physicians participated in the study. Among nurses, 2661 were female (90.3%), 636 were less than 31 years old (21.6%), 316 were short-term employees (10.7%), 1572 were full-time employees (53.4%), 1576 had specialisation or further education (53.5%) and 508 had less than 4 years of experience (17.2%). Among physicians, 287 were female (51.6%), 57 were less than 31 years old (10.3%), 244 were short-term employees (43.9%), 509 were full-time employees (91.5%), 351 had specialisation or further education (63.1%) and 112 had less than 4 years of experience (20.1%). Other demographic data are presented in Table 1.

### 3.2. Descriptive Statistics

Descriptive statistics for the different target groups are presented in Table 2. Competence development had the highest score among physicians (mean = 4.47, SD = 0.55) and nurses (mean = 4.45, SD = 0.53). Job performance had the second highest scores among physicians (mean = 4.05, SD = 0.46) and nurses (mean = 4.07, SD = 0.45). Uncertainty in patient treatment had the lowest score among physicians (mean = 1.84, SD = 0.40) and nurses (mean = 1.80, SD = 0.36). The statistical variation on the different indicators was generally considered satisfactory.

### 3.3. Variance across Sub-Groups

The results of MANOVA (Wilks’ lambda) indicated that age, gender and personnel category (physician or nurse) were significantly (*p* < 0.05) related to the variance of the seven dimensions included in Table 1. Hence, results generally indicated different scorings based on age, gender and personnel category. This indicates statistical variance and distribution, which is in accordance with expectancies in social science studies [48,49].

### 3.4. Correlations

In general, HMQ, LLQ, competence development, autonomy, colleague support and job performance were positively correlated with one another. On the other hand, uncertainty was negatively correlated with all the other dimensions, which is not unexpected. All correlations and Cronbach’s alpha can be seen in Table 3. Generally, all correlations were significant (*p* < 0.01), and the level of overlaps between concepts and internal consistency (Cronbach’s alpha) are considered satisfactory.

Ad hoc assessments were conducted to control for the different demographic variables listed in Table 1. Among the demographic variables, age had the strongest correlation with the concepts included in the study, and uncertainty was most strongly correlated with age among nurses (r = −0.23, *p* < 0.01) and physicians (r = −0.19, *p* < 0.01).

### 3.5. Confirmatory Factor Analyses

Confirmatory factor analyses (CFA) was performed using maximum likelihood extraction (MLE) to assess the validity of all concepts among nurses and physicians. All dimensions with associated items were included in the assessments (Table 4). CFA supported the use of the measurement concepts among nurses (IFI = 0.93, TLI = 0.91, CFI = 0.93, RMSEA = 0.049, 90% confidence interval = 0.047–0.051) and physicians (IFI = 0.93, TLI = 0.91, CFI = 0.93, RMSEA = 0.050, 90% confidence interval = 0.046–0.055). Among nurses, the standardised factor to item loadings ranged from 0.34–0.90, and among physicians loading ranged from 0.33–0.90. Uncertainty had the lowest loadings on “uncertainty regarding the use and function of special equipment”, which was 0.34 and 0.33 among nurses and physicians, respectively. Additionally, the loading on the items “Doubt if a patient’s relatives should get informed about the patient’s medical condition and treatment” was 0.37 among physicians. Even though two items had factor loading below 0.44, these items were not removed because the content of the items was relevant for capturing the theoretical domains they measure. Moreover, the theoretical concepts were operationalised with relatively few items, and none of the items were, therefore, considered redundant. Based on the fit indices and the overall results, the factor-to-item relations were considered satisfactory, indicating robust and valid measures. Thus, the structural model could be tested with the use of validated measurement concepts.

### 3.6. Results of Structural Equation Modelling (SEM)

**Figure 2:**
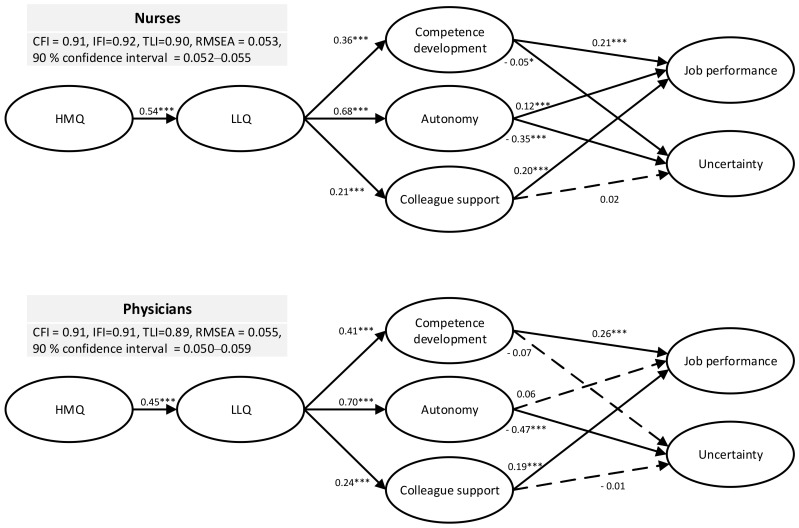
Structural modelling conducted on nurses and physicians. Note: * *p* < 0.05, *** *p* < 0.001. HMQ: Hospital management quality; LLQ: Local leadership quality.

The full structural model was tested (MLE) with all the latent and manifest variables. Among nurses, all fit indicators (Figure 2) were acceptable and above recommended thresholds. All beta coefficients were in the expected directions. HMQ was positively related to LLQ (b = 0.54, *p* < 0.001), supporting hypothesis 1. Further, LLQ was significantly related to competence development (b = 0.36, *p* < 0.001), colleague support (b = 0.21, *p* < 0.001) and autonomy (b = 0.68), supporting hypothesis 2. Additionally, job performance was significantly related to competence development (b = 0.21, *p* < 0.001), colleague support (b = 0.20, *p* < 0.001) and autonomy (b = 0.12, *p* < 0.001), as specified in hypothesis 3. Lastly, also supporting hypothesis 3, uncertainty was related to competence development (b = −0.05, *p* < 0.05) and autonomy (b = −0.35, *p* < 0.001), but not with colleague support (b = 0.02, *p* = not significant). Hence, the majority of the relations were significant and in expected directions, supporting the theoretical model among nurses. In total, the model explained 12% of the variance related to job performance, and 14% of the variance related to uncertainty among nurses.

The model fit was also considered adequate among physicians (Figure 2) even though TLI (0.89) was marginally below the 0.90 threshold. Further, all beta coefficients were in the expected directions. HMQ was positively related to LLQ (b = 0.45, < 0.001), supporting hypothesis 1. LLQ was significantly related to competence development (b = 0.41, *p* < 0.001), colleague support (b = 0.24, *p* < 0.001) and autonomy (b = 0.70, < 0.001), supporting hypothesis 2. According to hypothesis 3, psychological needs should be positively related to job performance and negatively related to uncertainty. Findings revealed that colleague support (b = 0.19, *p* < 0.001) and competence development (b = 0.26, *p* < 0.001) were positively related to job performance. Furthermore, autonomy was negatively related to uncertainty (b = −0.47, *p* < 0.001). Some non-significant results were also revealed among physicians. Autonomy was not significantly related to job performance, and uncertainty was not significantly related to competence development and colleague support. Among physicians, the model explained 13% of the variance related to job performance and 24% of the uncertainty.

Ad hoc assessments were conducted to control for the influence of age on uncertainty and job performance. Results revealed that age was significantly and negatively related to uncertainty both among nurses (b = −0.13, *p* < 0.001) and physicians (b = −0.13, *p* < 0.05) but did not influence job performance. However, the inclusion of age in the model reduced the model fit below the recommended thresholds and was, therefore, not included in the final model and presentation of the results (Figure 2).

## 4. Discussion

Overall, the majority of the results supported the theoretical framework proposed in this study: (1) HMQ is positively related to the quality of local leadership; (2) LLQ is positively related to psychological needs; (3) the majority of the psychological needs are positively related to job performance and negatively related to uncertainty. Moreover, the model is relevant and explains a substantial portion of variance related to uncertainty and job performance among nurses and physicians.

### 4.1. Theoretical and Practical Implications

Daily care of patients in hospital wards, typically provided by physicians, nurses and other hospital staff, depends on many factors that constitute the microsystems in hospitals. The working conditions of these employees, as well as their needs satisfaction in hospital microsystems are influenced by local managers, as suggested by the theoretical model developed in the current study. Furthermore, the model developed and tested in this study illustrates how HMQ can positively influence LLQ. Sometimes, this perspective is referred to as a multilevel organisational approach since top-level management has a responsibility to define organisational goals and strategies [50,51], which, in turn, influence local leaders and staff. In summary, this study suggests that line management, including both the hospital top-management and local leaders, have a significant and positive influence on psychological need satisfaction of both nurses and physicians. Moreover, the theoretical model developed in this study demonstrates that increased psychological need satisfaction will improve job performance and reduce uncertainty among nurses and physicians.

Managers develop and influence budgets, staff-to-patient ratios and opportunities for staff to develop competence through courses, learning activities and other initiatives. The approach used in the current study suggests multilevel organisational paths, indicating that managers indirectly influence the psychological needs of hospital employees while local leaders influence these needs directly. Since the fulfilment of psychological needs is expected to have a direct influence on both job performance and uncertainty, hospital top managers and local leaders play important roles in developing well-functioning and efficient microsystems, which take into consideration the psychological needs of physicians and nurses.

Hospital systems are hierarchical and complex structures comprising separate but interconnected components. The components of organisations are supposed to play complementary roles to accomplish their joint tasks [52,53,54]. However, shifts and different working hours make hospital teams and systems less stable, with the rotation of staff, individuals and patients [55]. Other barriers and challenges can be related to temporal resources, negative peer opinion, legislative hindrances and reimbursement shortfalls [56]. Additionally, complex organisations usually have competing agendas, values and goals [57,58] that may lead to fragmentation, competition and malpractice instead of integration, collaboration and cooperation between different actors of the system [58].

With different challenges and barriers towards providing quality care [56], the results of the current study suggest that hospital top-management and local leaders play crucial roles in adequately meeting the psychological needs of staff. Across sub-samples consisting of nurses and physicians, this finding is substantial. Local leaders strongly influence the competence development, autonomy and colleague support, although autonomy appears to have the strongest relation with LLQ across the sub-groups. Interestingly, the relation between autonomy and job performance is not significant among physicians. Autonomy might be related to more advanced and difficult tasks, which may explain why colleague support and competence development are generally more significantly related to job performance. Hence, local leaders and work process designers should be aware that higher levels of autonomy do not necessarily provide higher levels of job performance among physicians. Moreover, competence development and support from colleagues might have a more positive influence on job performance, which implies that local leaders should emphasise competence development and support from colleagues to improve wards and clinical working conditions. However, nurses’ autonomy significantly influences job performance. As a general approach, increasing autonomy among nurses seems to be an adequate strategy. However, the results suggest that colleague support and competence development are more important when trying to improve job performance among physicians.

Research and literature [55,59,60] emphasise that managers and leaders need to establish integrated care and address other challenges, such as the development of strong patient safety cultures [61]. According to the theoretical model developed and examined in the current study, the fulfilment of the psychological needs of nurses and physicians may be more important in the development of integrated care and patient safety cultures than earlier assumed and emphasised in research. As such, the findings suggest that attention to psychological need satisfaction among hospital staff can or should be higher on the agenda when developing leadership development programs in hospitals. Further, the fulfilment of psychological needs should be incorporated and emphasised in hospital improvement programs and the design of the working conditions for hospital staff.

### 4.2. Research Limitations

The findings of this study should be considered in view of the following limitations. The sample size comprising nurses (*N* = 2946) was much greater compared to that of physicians (*N* = 556). Nevertheless, the statistical power should be considered relatively robust in the physician sample. Future studies, however, should aim at using large samples to reduce the likelihood of type II error. Moreover, this study is conducted using a sample of physicians and nurses in a single country, and more studies should be conducted in other cultures and contexts before the generalisation of the results. The cross-sectional design incorporates staff perception and does not establish evidence of causal relationships. Hence, other types of research design, analysing multiple time periods, are suggested for follow-up studies. In addition, future studies can consider the use of different types of job performance indicators, as job performance is a difficult concept to measure [62].

## 5. Conclusions

The results suggest hospitals and their line managers should aim at increasing job performance and reducing uncertainty by fulfilling the psychological needs of their employees. The results suggest that psychological needs perspectives should be integrated into quality-improvement interventions and strategies for nurses and physicians. The findings illustrate how hospital line managers need to address the psychological needs of nurses and physicians in hospital settings. For health professionals to be efficient and confident in what they do, the social environment must provide experiences that will satisfy their basic psychological needs [63]. The results of the current study suggest that the job performance of hospital staff will suffer without psychological nutrients, and because of this, patients are likely to suffer as well. The hospitals’ HR practices should incorporate perspectives related to the satisfaction of workers’ psychological needs in the development of managers while considering job characteristics and organisational systems. To be able to deliver high quality care, the psychological needs of nurses and physicians must be met. Achieving a fit between individuals and their jobs is an important approach to increase psychological need satisfaction.

## Figures and Tables

**Table 1:** Participants in the study.

Demographic Variables		Physicians (*N* = 556)		Nurses (*N* = 2946)			Total Sample (*N* = 3502)
		*n*	%	*n*	%	*n*	%
Gender							
	Female	287	51.6	2661	90.3	2948	84.2
	Male	269	48.4	285	9.7	554	15.8
Age							
	<31	57	10.3	636	21.6	693	19.8
	31–40	243	43.7	753	25.6	996	28.4
	41–50	130	23.4	749	25.4	879	25.1
	51–60	80	14.4	651	22.1	731	20.9
	>60	46	8.3	157	5.3	203	5.8
Employment (long-/short-term)							
	Long-term employee	312	56.1	2630	89.3	2942	84.0
	Short-term employee	244	43.9	316	10.7	560	16.0
Employment (full-/part-time)							
	Full-time employee	509	91.5	1572	53.4	2081	59.4
	Part-time employee	47	8.5	1374	46.6	1421	40.6
Specialisation/further education							
	Yes	351	63.1	1576	53.5	1927	55.0
	No	205	36.9	1370	46.5	1575	45.0
Years of experience							
	≤4	112	20.1	508	17.2	620	21.7
	5–10	140	25.2	512	17.4	652	22.8
	11–20	117	21.0	709	24.1	826	28.9
	≥21	95	17.1	668	22.7	763	26.7

**Table 2:** Descriptive statistics.

	Physicians		Nurses		Total	
	Mean	SD	Mean	SD	Mean	SD
Hospital management quality (HMQ)	3.00	0.87	3.11	0.79	3.10	0.80
Local leadership quality (LLQ)	3.75	1.01	3.75	0.97	3.75	0.97
Autonomy	3.07	0.87	3.24	0.79	3.21	0.81
Competence development	4.47	0.55	4.45	0.53	4.45	0.53
Colleague support	3.52	0.70	3.47	0.67	3.48	0.67
Job performance	4.05	0.46	4.07	0.45	4.07	0.46
Uncertainty	1.84	0.40	1.80	0.36	1.81	0.37

**Table 3:** Cronbach’s alpha (total sample in parentheses) and correlations among nurses (below diagonal) and physicians (above diagonal).

	1	2	3	4	5	6	7
1. Hospital management quality (HMQ)	(0.84)	0.42	0.23	0.48	0.11	0.19	−0.54
2. Local leadership quality (LLQ)	0.52	(0.86)	0.39	0.67	0.22	0.24	−0.44
3. Competence development	0.18	0.33	(0.70)	0.35	0.23	0.30	−0.20
4. Autonomy	0.43	0.66	0.32	(0.91)	0.27	0.18	−0.49
5. Colleague support	0.12	0.18	0.32	0.24	(0.74)	0.25	−0.14
6. Job performance	0.21	0.21	0.29	0.22	0.28	(0.77)	−0.30
7. Uncertainty	−0.43	−0.39	−0.12	−0.36	−0.06	−0.29	(0.65)

Note: All correlations are significant at *p* < 0.001 (two-tailed test).

**Table 4:** Standardised factor loadings based on confirmatory factor analyses (CFA).

Dimension/Item	Standardised Factor Loadings	
	Nurses	Physicians
Competence development		
Does your work require you to change?	0.63	0.55
Does your work require you to take initiative?	0.64	0.60
Can you use your skills and your expertise in your job?	0.68	0.76
Do you have the opportunity to learn new things through your work?	0.68	0.75
Autonomy		
In my department, we often influence goals or measures	0.87	0.86
Employees have the possibility to influence the work situations	0.89	0.90
All of the employees in my department are involved in important decisions that affect them	0.85	0.87
In my department, we can influence requirements associated with doing a good job	0.80	0.78
Colleague support		
Give your colleagues constructive advice	0.74	0.72
Do your colleagues express their opinions about your work?	0.81	0.79
Are your colleagues able to appreciate the value of your work and see the results of it?	0.58	0.57
Local leadership quality (LLQ)		
The leader of my unit emphasises the development of employees	0.84	0.90
My nearest leader has good knowledge about my work situation	0.71	0.67
The leader of my unit emphasises keeping employees	0.86	0.89
Hospital management quality (HMQ)		
The hospital management has good knowledge about the situation in the departments	0.79	0.85
In my hospital, the management priorities are correctly based on a holistic understanding	0.90	0.88
The regional hospital management has good knowledge of our hospital	0.73	0.73
Uncertainty		
Insufficient information from other healthcare professionals regarding a patient’s medical condition	0.52	0.42
Providing wrong treatment to a patient	0.54	0.43
No doctor present at a medical emergency	0.46	0.44
Too few personnel to provide reasonable treatment	0.58	0.65
Doubt if a patient’s relatives should be informed about the patient’s medical condition and treatment	0.48	0.37
Uncertainty regarding the use and function of special equipment	0.34	0.33
Job performance		
Are you satisfied with the quality of the work you perform?	0.80	0.70
Are you satisfied with the amount of work you get done?	0.78	0.60
Are you satisfied with your ability to resolve problems that pop up during your work?	0.70	0.79
Are you satisfied with your ability to have a good relationship with your work colleagues?	0.48	0.54

## Data Availability

Not applicable or agreed with the Western Norway Regional Health Authority.
